# Abuse of Calomel by Southern Physicians

**Published:** 1842-12

**Authors:** 


### ABUSE OF CALOMEL BY SOUTHERN PHYSICIANS.

The paragraph below has met our eye in several newspapers, and we believe it has quite made the circle of the Union. From what "lectures" it is taken, or how it got into the public prints, we are entirely ignorant.

"The following extract is from the lectures of N. Chapman, M. D., Professor of the Institutes and Practice of Medicine in the University of Pennsylvania, located in Philadelphia. He thus discourseth on the use of calomel:

Gentlemen—If you could only see what I almost daily see in my private practice in this city, persons from the south, in the very last stages of a wretched existence, emaciated to a skeleton, with both tables of the skull almost completely perforated in many cases, the nose half gone, with rotten jaws, ulcerated throats, breaths more pestiferous, more intolerable than poisonous upas, limbs racked with the pain of the inquisition, mind as imbecile as the puling babe's, a grievous burden to themselves, and a disgusting spectacle to others, you would exclaim, as I have often done, "Oh! the lamentable want of science that dictates abuse of that noxious drug, calomel, in the southern States!"

A horrible catalogue, indeed! a very lazar-house picture, fearful enough to contemplate, and embellished with the acknowleged skill of the author, but which, nevertheless, displays rather his imagination than his knowledge of the actual state of things. That southern physicians do use calomel in larger doses than their northern brethren, is certainly true; but so do they use quinine, opium, and many other potent articles. The reason is obvious: the diseases with which they have to contend, are rapid and dangerous to a degree entirely unknown further north, and their remedial measures have to be pursued with an energy and decision that northern diseases seldom require. Dr. Chapman need not be told that the susceptibility to remedies varies with climate, &c., and that a dose of medicine which would be all-sufficient in Philadelphia, would be wholly insufficient in New Orleans.

Now it is a little singular that while such evils as the above are charged against calomel, we never hear any thing of bad consequences arising from the other powerful and poisonous drugs we have named. We believe they would be just as well founded in the latter case as they are in the former. And we do not believe they are in either. We do not believe that mercury, when given for other diseases than those of venereal origin, will be followed by the effects here depicted; and the experience of a majority of the profession bears us out in this non-belief. In never a single instance have such effects been seen, save where the individual had led a suspicious life. Take a man who has never had syphilis, and let him be *drugged* with calomel till you are tired, and he will not have perforations of the cranium, nor ulcerations of the nasal bones, nor nocturnal pains, nor any of the thousand and one affections that are ascribed to it.

Our southern friends will no doubt feel exceedingly obliged to Prof. Chapman for his high opinion of their intelligence and scientific attainments. "Oh! the lamentable want of science that dictates abuse of that noxious drug, calomel, in the southern States!" A most pathetic exclamation it is, truly! The rebuke comes, too, with an excellent grace from the man who lent his name to give currency to Swaim's *Panacea!*—a *nostrum* which has done incalculable harm, and to which these lamentable effects, if they exist, are in many cases to be ascribed. And the unction attending this objurgation is still more remarkable, when we recollect that the physicians thus aspersed, received their medical education at the hands of the learned professor himself. It is a well-known fact that a very large majority of southern physicians are graduates of the University of Pennsylvania, and it is to be presumed that they use calomel and other articles as they were there taught to use them.

Granting, though, that this picture, with all its Titian-like coloring, is strictly true, there are other modes of expressing the same truth, not so obnoxious to censure. We go for telling the truth always, be it remembered, cut where it will; but the mode, the manner, may be a fair subject of consideration. Now we object to the mode here pursued, apart from the rank injustice it does to a numerous and highly respectable portion of the profession. The advocates of the various quack medicines and systems paraded through the country, will catch at this extract, as if it were a very god-send; changes will be rung upon it, and it will be emblazoned every where, much to the detriment of society. Many a one will be frightened at such a fearful array of consequences, and refuse, may be when life itself depends upon it, the employment of the indispensable drug. Would Dr. Chapman end his career as he began it, by playing into the hands of the veriest charlatans that ever went unwhipped of justice? We fain hope that no such sentiments have been expressed by him, and that the paragraph we have quoted is a forgery. C.

